# *N*-(2-Fluoro­phen­yl)-2,6-dimethyl-1,3-dioxan-4-amine

**DOI:** 10.1107/S1600536813024732

**Published:** 2013-09-12

**Authors:** Zeenat Fatima, Gottimukkala Rambabu, Bandapalli Palakshi Reddy, Vijayaparthasarathi Vijayakumar, Devadasan Velmurugan

**Affiliations:** a Centre of Advanced Study in Crystallography and Biophysics, University of Madras, Guindy Campus, Chennai 600 025, India; b Chemistry Department, GEBH, Sree Vidyanikethan Engineering College, A. Rangampet, Tirupati 517102, India; c Centre for Organic and Medicinal Chemistry, VIT University, Vellore 632 014, India

## Abstract

In the title compound, C_12_H_16_FNO_3_, the dioxane ring adopts a chair conformation with the methyl groups and amine N atom in equatorial positions. The best plane through the dioxane ring makes a dihedral angle of 43.16 (8)° with the phenyl ring. In the crystal, pairs of C—H⋯O hydrogen bonds link the mol­ecules into centrosymmetric *R*_2_^2^(8) dimers, which are linked into [100] chains by further C—H⋯O hydrogen bonds. The N—H group does not participate in hydrogen bonding.

## Related literature

Dioxane rings are frequently encountered in structural motifs in many bioactive mol­ecules such as cytotoxic agents (Aubele *et al.*, 2005 ▶) and anti­muscarinic agents (Marucci *et al.*, 2005 ▶). For applications of this class of compounds, see: Wang, Yuan, Liu *et al.* (1996 ▶); Wang, Yuan, Lei & Liu (1996 ▶); Yuan *et al.* (2005 ▶). For related crystal structures, see: Chuprunov *et al.* (1981 ▶).
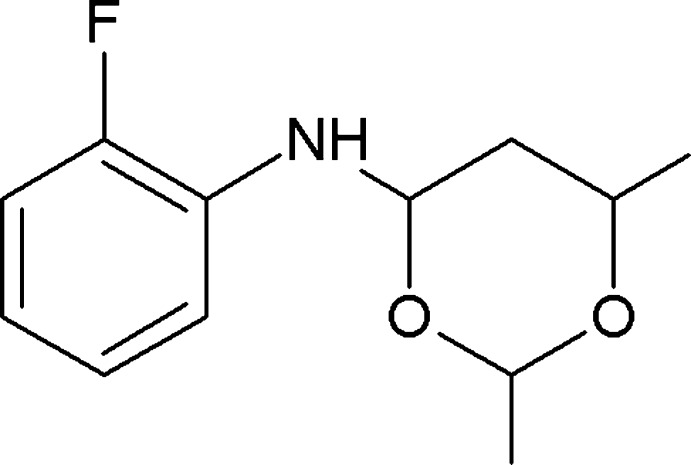


## Experimental

### 

#### Crystal data

C_12_H_16_FNO_2_
*M* *_r_* = 225.26
Monoclinic, 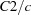

*a* = 19.6219 (13) Å
*b* = 8.1603 (6) Å
*c* = 15.2396 (10) Å
β = 95.950 (3)°
*V* = 2427.0 (3) Å^3^
*Z* = 8
Mo *K*α radiation
μ = 0.09 mm^−1^
*T* = 293 K
0.25 × 0.20 × 0.15 mm

#### Data collection

Bruker SMART APEXII CCD diffractometer
Absorption correction: multi-scan (*SADABS*; Bruker, 2008 ▶) *T*_min_ = 0.617, *T*_max_ = 0.746
11540 measured reflections
3020 independent reflections
1973 reflections with *I* > 2σ(*I*)
*R*_int_ = 0.026

#### Refinement

*R*[*F* ^2^ > 2σ(*F* ^2^)] = 0.044
*wR*(*F* ^2^) = 0.137
*S* = 1.03
3020 reflections
153 parameters
H atoms treated by a mixture of independent and constrained refinement
Δρ_max_ = 0.18 e Å^−3^
Δρ_min_ = −0.23 e Å^−3^

### 

Data collection: *APEX2* (Bruker, 2008 ▶); cell refinement: *SAINT* (Bruker, 2008 ▶); data reduction: *SAINT*; program(s) used to solve structure: *SHELXS97* (Sheldrick, 2008 ▶); program(s) used to refine structure: *SHELXL97* (Sheldrick, 2008 ▶); molecular graphics: *ORTEP-3 for Windows* (Farrugia, 2012 ▶); software used to prepare material for publication: *SHELXL97* and *PLATON* (Spek, 2009 ▶).

## Supplementary Material

Crystal structure: contains datablock(s) global, I. DOI: 10.1107/S1600536813024732/hb7132sup1.cif

Structure factors: contains datablock(s) I. DOI: 10.1107/S1600536813024732/hb7132Isup2.hkl

Supplementary material file. DOI: 10.1107/S1600536813024732/hb7132Isup3.cml

Additional supplementary materials:  crystallographic information; 3D view; checkCIF report

## Figures and Tables

**Table 1:** Hydrogen-bond geometry (Å, °)

*D*—H⋯*A*	*D*—H	H⋯*A*	*D*⋯*A*	*D*—H⋯*A*
C5—H5*A*⋯O2^i^	0.97	2.60	3.5563 (17)	169
C11—H11⋯O1^ii^	0.93	2.54	3.473 (2)	177

Symmetry codes: (i) 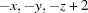; (ii) 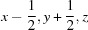.

**Fig. 1.**
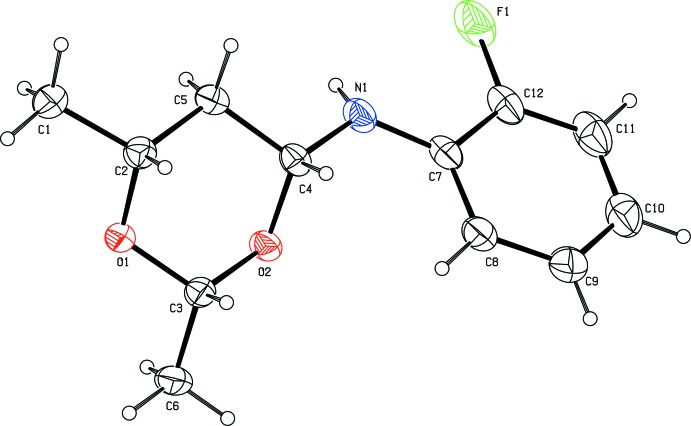
The molecular structure of the title compound, showing displacement ellipsoids drawn at the 30% probability level.

**Fig. 2.**
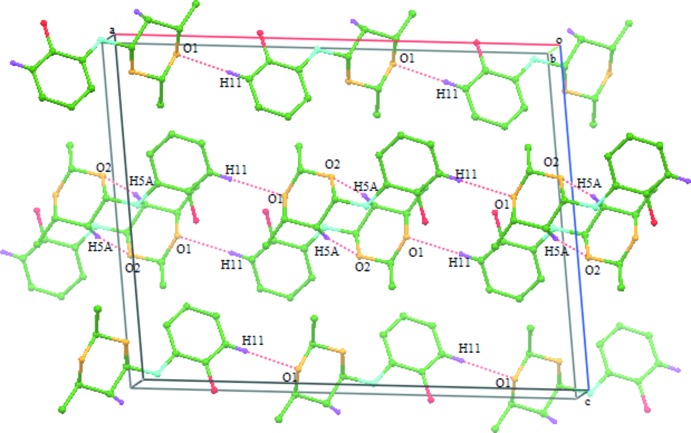
The crystal packing of the title compound viewed down b axis. H-atoms not involved in H-bonds have been excluded for clarity.

## supplementary crystallographic information

### 1. Comment

Oxygen heterocycles play a vital role as basic building blocks in pharmaceutical preparations. This class of compounds has useful insecticidal as well as anti-foaming properties (Yuan *et al.*, 2005). Dioxane rings are frequently encountered in structural motifs in many bioactive molecules such as cytotoxic agents (Aubele *et al.*, 2005) and antimuscarinic agents (Marucci *et al.*, 2005). As part of our studies in this area, we have undertaken a single-crystal structure determination of the title compound.

In the title compound, C_12_H_16_F_1_N_1_O_3_ (Fig.1), pairs of C—H···O hydrogen bonds link the molecules into centrosymmetric *R*_2_^2^(8) dimers (Fig.2). The dimers are linked into an infinite chain propagating along the 'a' axis by further C—H···O hydrogen bonds. The dioxane ring adopts a *chair* conformation and the best plane through the dioxane ring makes a dihedral angle of 43.16 (8)° with the phenyl ring.

### 2. Experimental

To 2-fluoroaniline (1 mmol), acetaldehyde (3 mmol) was added dropwise and stirred for about 4 h at 0 °C. The progress of the reaction was monitored through TLC. The reaction mixture was washed with petroleum ether. Resultant was dissolved in diethylether and allowed to evaporate. Solid product obtained was recrystallized for diethylether solution to yield colourless blocks.

### 3. Refinement

The hydrogen atoms were placed in calculated positions with C—H = 0.93 Å to 0.98 Å refined in the riding model with fixed isotropic displacement parameters:*U*_iso_(H) = 1.5*U*_eq_(C) for methyl group and *U*_iso_(H) = 1.2*U*_eq_(C) for other groups.

### Crystal data

**Table:**

C_12_H_16_FNO_2_	*F*(000) = 960
*M**_r_* = 225.26	*D*_x_ = 1.233 Mg m^−^^3^
Monoclinic, *C*2/*c*	Mo *K*α radiation, λ = 0.71073 Å
Hall symbol: -C 2yc	Cell parameters from 3020 reflections
*a* = 19.6219 (13) Å	θ = 2.7–28.4°
*b* = 8.1603 (6) Å	µ = 0.09 mm^−^^1^
*c* = 15.2396 (10) Å	*T* = 293 K
β = 95.950 (3)°	Block, colourless
*V* = 2427.0 (3) Å^3^	0.25 × 0.20 × 0.15 mm
*Z* = 8	

### Data collection

**Table:**

Bruker SMART APEXII CCD diffractometer	3020 independent reflections
Radiation source: fine-focus sealed tube	1973 reflections with *I* > 2σ(*I*)
Graphite monochromator	*R*_int_ = 0.026
ω and φ scans	θ_max_ = 28.4°, θ_min_ = 2.7°
Absorption correction: multi-scan (*SADABS*; Bruker, 2008)	*h* = −26→25
*T*_min_ = 0.617, *T*_max_ = 0.746	*k* = −10→10
11540 measured reflections	*l* = −17→20

### Refinement

**Table:**

Refinement on *F*^2^	Primary atom site location: structure-invariant direct methods
Least-squares matrix: full	Secondary atom site location: difference Fourier map
*R*[*F*^2^ > 2σ(*F*^2^)] = 0.044	Hydrogen site location: inferred from neighbouring sites
*wR*(*F*^2^) = 0.137	H atoms treated by a mixture of independent and constrained refinement
*S* = 1.03	*w* = 1/[σ^2^(*F*_o_^2^) + (0.0629*P*)^2^ + 0.5739*P*] where *P* = (*F*_o_^2^ + 2*F*_c_^2^)/3
3020 reflections	(Δ/σ)_max_ = 0.001
153 parameters	Δρ_max_ = 0.18 e Å^−^^3^
0 restraints	Δρ_min_ = −0.23 e Å^−^^3^

### Special details

**Table:**

Geometry. All e.s.d.'s (except the e.s.d. in the dihedral angle between two l.s. planes) are estimated using the full covariance matrix. The cell e.s.d.'s are taken into account individually in the estimation of e.s.d.'s in distances, angles and torsion angles; correlations between e.s.d.'s in cell parameters are only used when they are defined by crystal symmetry. An approximate (isotropic) treatment of cell e.s.d.'s is used for estimating e.s.d.'s involving l.s. planes.
Refinement. Refinement of *F*^2^ against ALL reflections. The weighted *R*-factor *wR* and goodness of fit *S* are based on *F*^2^, conventional *R*-factors *R* are based on *F*, with *F* set to zero for negative *F*^2^. The threshold expression of *F*^2^ > σ(*F*^2^) is used only for calculating *R*-factors(gt) *etc*. and is not relevant to the choice of reflections for refinement. *R*-factors based on *F*^2^ are statistically about twice as large as those based on *F*, and *R*- factors based on ALL data will be even larger.

### Fractional atomic coordinates and isotropic or equivalent isotropic displacement parameters (Å^2^)

**Table:**

	*x*	*y*	*z*	*U*_iso_*/*U*_eq_	
H1	−0.0550 (9)	0.220 (2)	1.0096 (12)	0.074 (5)*	
H3	0.1172 (8)	0.1912 (19)	0.8533 (10)	0.058 (4)*	
O1	0.13369 (5)	0.02783 (12)	0.94558 (6)	0.0592 (3)	
O2	0.02809 (4)	0.13036 (11)	0.88947 (6)	0.0535 (3)	
C4	0.02808 (7)	0.25397 (16)	0.95733 (9)	0.0526 (3)	
H4	0.0476	0.3552	0.9360	0.063*	
C5	0.07197 (7)	0.19735 (17)	1.03878 (9)	0.0545 (3)	
H5A	0.0500	0.1056	1.0649	0.065*	
H5B	0.0767	0.2854	1.0817	0.065*	
N1	−0.04050 (7)	0.28505 (17)	0.97514 (9)	0.0617 (3)	
C2	0.14205 (7)	0.14617 (18)	1.01572 (9)	0.0561 (3)	
H2	0.1657	0.2425	0.9953	0.067*	
C3	0.09501 (7)	0.0917 (2)	0.87025 (9)	0.0562 (4)	
C7	−0.08824 (7)	0.35496 (17)	0.91321 (10)	0.0598 (4)	
F1	−0.16772 (6)	0.33920 (15)	1.01790 (9)	0.1024 (4)	
C6	0.09070 (10)	−0.0353 (3)	0.79920 (11)	0.0828 (6)	
H6A	0.1360	−0.0620	0.7852	0.124*	
H6B	0.0642	0.0064	0.7475	0.124*	
H6C	0.0691	−0.1320	0.8192	0.124*	
C12	−0.15451 (8)	0.3829 (2)	0.93551 (13)	0.0723 (5)	
C1	0.18616 (9)	0.0692 (2)	1.09141 (10)	0.0745 (5)	
H1A	0.2296	0.0394	1.0725	0.112*	
H1B	0.1639	−0.0270	1.1108	0.112*	
H1C	0.1931	0.1460	1.1392	0.112*	
C8	−0.07505 (9)	0.4054 (2)	0.83014 (11)	0.0733 (5)	
H8	−0.0318	0.3885	0.8120	0.088*	
C9	−0.12519 (11)	0.4808 (2)	0.77326 (12)	0.0899 (6)	
H9	−0.1151	0.5158	0.7180	0.108*	
C11	−0.20465 (10)	0.4542 (3)	0.88042 (17)	0.0926 (7)	
H11	−0.2482	0.4692	0.8979	0.111*	
C10	−0.18988 (12)	0.5039 (3)	0.79822 (16)	0.0989 (7)	
H10	−0.2236	0.5532	0.7596	0.119*	

### Atomic displacement parameters (Å^2^)

**Table:**

	*U*^11^	*U*^22^	*U*^33^	*U*^12^	*U*^13^	*U*^23^
O1	0.0587 (6)	0.0740 (6)	0.0451 (5)	0.0204 (5)	0.0063 (4)	−0.0013 (4)
O2	0.0435 (5)	0.0652 (5)	0.0531 (5)	0.0060 (4)	0.0112 (4)	−0.0052 (4)
C4	0.0477 (8)	0.0510 (7)	0.0608 (8)	0.0028 (6)	0.0144 (6)	−0.0025 (6)
C5	0.0596 (9)	0.0530 (7)	0.0523 (8)	0.0005 (6)	0.0128 (6)	−0.0070 (6)
N1	0.0521 (7)	0.0665 (7)	0.0695 (8)	0.0106 (6)	0.0208 (6)	−0.0006 (7)
C2	0.0533 (8)	0.0627 (8)	0.0526 (8)	0.0015 (6)	0.0068 (6)	0.0016 (6)
C3	0.0474 (8)	0.0767 (9)	0.0461 (7)	0.0128 (7)	0.0113 (6)	0.0061 (7)
C7	0.0516 (8)	0.0563 (7)	0.0721 (10)	0.0061 (6)	0.0093 (7)	−0.0185 (7)
F1	0.0756 (7)	0.1085 (8)	0.1316 (10)	0.0213 (6)	0.0513 (7)	0.0091 (7)
C6	0.0734 (11)	0.1247 (15)	0.0507 (8)	0.0338 (10)	0.0076 (8)	−0.0165 (9)
C12	0.0542 (9)	0.0690 (9)	0.0948 (12)	0.0079 (7)	0.0129 (9)	−0.0202 (9)
C1	0.0746 (11)	0.0911 (11)	0.0554 (9)	0.0164 (9)	−0.0043 (8)	−0.0047 (8)
C8	0.0689 (10)	0.0814 (10)	0.0697 (10)	0.0192 (8)	0.0081 (8)	−0.0148 (8)
C9	0.1005 (16)	0.0957 (13)	0.0703 (11)	0.0248 (11)	−0.0069 (10)	−0.0226 (10)
C11	0.0544 (10)	0.0973 (13)	0.1236 (18)	0.0145 (9)	−0.0033 (11)	−0.0348 (13)
C10	0.0798 (14)	0.1034 (14)	0.1047 (16)	0.0290 (11)	−0.0316 (12)	−0.0404 (13)

### Geometric parameters (Å, º)

**Table:**

O1—C3	1.4087 (16)	C7—C12	1.396 (2)
O1—C2	1.4373 (17)	F1—C12	1.356 (2)
O2—C3	1.4105 (16)	C6—H6A	0.9600
O2—C4	1.4446 (16)	C6—H6B	0.9600
C4—N1	1.4228 (17)	C6—H6C	0.9600
C4—C5	1.508 (2)	C12—C11	1.357 (3)
C4—H4	0.9800	C1—H1A	0.9600
C5—C2	1.513 (2)	C1—H1B	0.9600
C5—H5A	0.9700	C1—H1C	0.9600
C5—H5B	0.9700	C8—C9	1.386 (2)
N1—C7	1.382 (2)	C8—H8	0.9300
N1—H1	0.820 (18)	C9—C10	1.375 (3)
C2—C1	1.506 (2)	C9—H9	0.9300
C2—H2	0.9800	C11—C10	1.376 (3)
C3—C6	1.495 (2)	C11—H11	0.9300
C3—H3	0.969 (16)	C10—H10	0.9300
C7—C8	1.381 (2)		
			
C3—O1—C2	111.59 (11)	C8—C7—C12	116.24 (16)
C3—O2—C4	111.98 (10)	N1—C7—C12	118.92 (15)
N1—C4—O2	109.35 (11)	C3—C6—H6A	109.5
N1—C4—C5	111.61 (11)	C3—C6—H6B	109.5
O2—C4—C5	109.44 (10)	H6A—C6—H6B	109.5
N1—C4—H4	108.8	C3—C6—H6C	109.5
O2—C4—H4	108.8	H6A—C6—H6C	109.5
C5—C4—H4	108.8	H6B—C6—H6C	109.5
C4—C5—C2	110.40 (11)	F1—C12—C11	119.33 (17)
C4—C5—H5A	109.6	F1—C12—C7	117.05 (16)
C2—C5—H5A	109.6	C11—C12—C7	123.6 (2)
C4—C5—H5B	109.6	C2—C1—H1A	109.5
C2—C5—H5B	109.6	C2—C1—H1B	109.5
H5A—C5—H5B	108.1	H1A—C1—H1B	109.5
C7—N1—C4	122.05 (13)	C2—C1—H1C	109.5
C7—N1—H1	116.8 (13)	H1A—C1—H1C	109.5
C4—N1—H1	113.9 (13)	H1B—C1—H1C	109.5
O1—C2—C1	107.55 (12)	C7—C8—C9	121.15 (18)
O1—C2—C5	108.78 (11)	C7—C8—H8	119.4
C1—C2—C5	113.66 (12)	C9—C8—H8	119.4
O1—C2—H2	108.9	C10—C9—C8	120.2 (2)
C1—C2—H2	108.9	C10—C9—H9	119.9
C5—C2—H2	108.9	C8—C9—H9	119.9
O1—C3—O2	110.36 (10)	C12—C11—C10	118.84 (19)
O1—C3—C6	108.61 (13)	C12—C11—H11	120.6
O2—C3—C6	108.76 (13)	C10—C11—H11	120.6
O1—C3—H3	107.9 (9)	C9—C10—C11	119.91 (19)
O2—C3—H3	108.7 (9)	C9—C10—H10	120.0
C6—C3—H3	112.5 (9)	C11—C10—H10	120.0
C8—C7—N1	124.81 (14)		
			
C3—O2—C4—N1	−178.72 (11)	C4—N1—C7—C8	−1.8 (2)
C3—O2—C4—C5	−56.18 (14)	C4—N1—C7—C12	−179.59 (13)
N1—C4—C5—C2	173.31 (11)	C8—C7—C12—F1	−177.83 (14)
O2—C4—C5—C2	52.12 (15)	N1—C7—C12—F1	0.2 (2)
O2—C4—N1—C7	−65.93 (17)	C8—C7—C12—C11	0.4 (2)
C5—C4—N1—C7	172.83 (12)	N1—C7—C12—C11	178.40 (16)
C3—O1—C2—C1	−177.76 (12)	N1—C7—C8—C9	−177.28 (16)
C3—O1—C2—C5	58.73 (14)	C12—C7—C8—C9	0.6 (2)
C4—C5—C2—O1	−53.33 (14)	C7—C8—C9—C10	−1.2 (3)
C4—C5—C2—C1	−173.11 (12)	F1—C12—C11—C10	177.44 (17)
C2—O1—C3—O2	−62.98 (16)	C7—C12—C11—C10	−0.7 (3)
C2—O1—C3—C6	177.87 (13)	C8—C9—C10—C11	0.9 (3)
C4—O2—C3—O1	61.46 (15)	C12—C11—C10—C9	0.1 (3)
C4—O2—C3—C6	−179.49 (12)		

### Hydrogen-bond geometry (Å, º)

**Table:**

*D*—H···*A*	*D*—H	H···*A*	*D*···*A*	*D*—H···*A*
C5—H5*A*···O2^i^	0.97	2.60	3.5563 (17)	169
C11—H11···O1^ii^	0.93	2.54	3.473 (2)	177

Symmetry codes: (i) −*x*, −*y*, −*z*+2; (ii) *x*−1/2, *y*+1/2, *z*.
